# Pseudotyping Bacteriophage P2 Tail Fibers to Extend the Host Range for Biomedical Applications

**DOI:** 10.1021/acssynbio.1c00629

**Published:** 2022-09-09

**Authors:** Tabitha G. Cunliffe, Alan L. Parker, Alfonso Jaramillo

**Affiliations:** † Division of Cancer and Genetics, School of Medicine, Cardiff University, Heath Park, Cardiff CF14 4XN, U.K.; ‡ School of Life Sciences, University of Warwick, Coventry CV4 7AL, U.K.; § Systems Immunity University Research Institute, School of Medicine, Cardiff University, Heath Park, Cardiff CF14 4XN, U.K.; ∥ De Novo Synthetic Biology Laboratory, I2SysBio, CSIC-University of Valencia, Parc Científic Universitat de València, Calle Catedrático Agustín Escardino, 9, 46980 Paterna, Spain

**Keywords:** bacteriophage, pseudotyping, chimera, retargeting, tropism, antimicrobial resistance

## Abstract

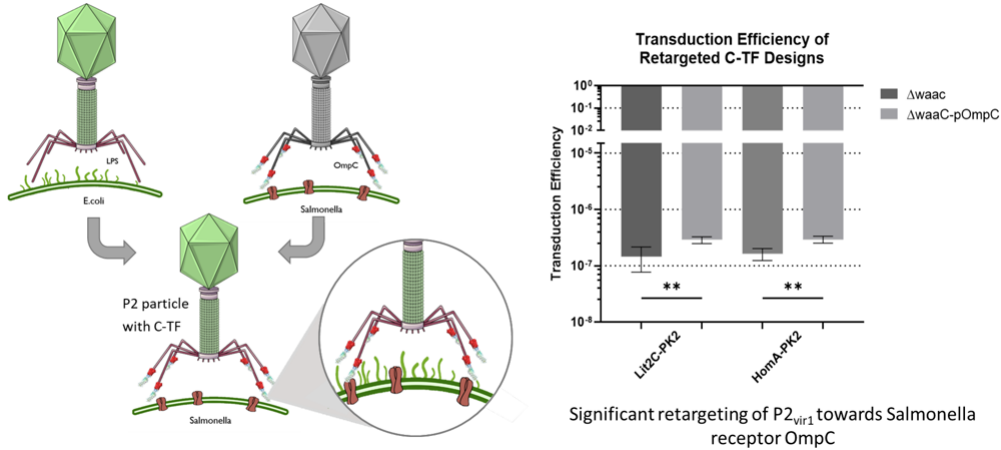

Bacteriophages (phages) represent powerful potential treatments against antibiotic-resistant bacterial infections. Antibiotic-resistant bacteria represent a significant threat to global health, with an estimated 70% of infection-causing bacteria being resistant to one or more antibiotics. Developing novel antibiotics against the limited number of cellular targets is expensive and time-consuming, and bacteria can rapidly develop resistance. While bacterial resistance to phage can evolve, bacterial resistance to phage does not appear to spread through lateral gene transfer, and phage may similarly adapt through mutation to recover infectivity. Phages have been identified for all known bacteria, allowing the strain-selective killing of pathogenic bacteria. Here, we re-engineered the *Escherichia coli* phage P2 to alter its tropism toward pathogenic bacteria. Chimeric tail fibers formed between P2 and S16 genes were designed and generated through two approaches: homology- and literature-based. By presenting chimeric P2:S16 fibers on the P2 particle, our data suggests that the resultant phages were effectively detargeted from the native P2 cellular target, lipopolysaccharide, and were instead able to infect via the proteinaceous receptor, OmpC, the natural S16 receptor. Our work provides evidence that pseudotyping P2 is feasible and can be used to extend the host range of P2 to alternative receptors. Extension of this work could produce alternative chimeric tail fibers to target pathogenic bacterial threats. Our engineering of P2 allows adsorption through a heterologous outer-membrane protein without culturing in its native host, thus providing a potential means of engineering designer phages against pathogenic bacteria from knowledge of their surface proteome.

## 1 Introduction

Bacteriophages have various advantages over traditional antibiotics. In nature, there are an estimated 10^31^ phage particles,^1^ and thus it is theoretically likely that there is a phage capable of infecting every strain of bacteria on the planet. Phages can be incredibly specific, unlike traditional antibiotics, which are effective against a large spectrum of bacteria. This pinpoint specificity is a significant advantage, as it removes any chance of other bacteria present in the patient developing resistance to the treatment and eliminates side effects on beneficial bacteria.^2,3^ Moreover, phages only replicate in target bacterial cells and cannot infect mammalian cells, as shown in the experimental work in humans where no adverse symptoms were reported.^4^

Antibiotic-resistant bacteria represent one of the biggest threats to global health, according to the World Health Organization (WHO),^5^ with estimates suggesting that around 70% of bacteria that cause infections are resistant to one or more antibiotics.^6^ The development of new traditional antibiotics has shown limited success due to the incredibly long time from research to market and the very high cost of development.^7^ Additionally, bacteria rapidly mutate to evolve resistance to new antibiotics. For example, the new antibiotic Ceftaroline was introduced in 2010 in the United States and approved in 2012 by the European Commission.^8^ However, in just 1 year from its introduction, resistance to Ceftaroline was discovered in clinical samples of *Staphylococcus*.^9^ Currently, without resistance to new agents, there are not sufficient novel drugs in the development process to cope with the current burden of antibiotic resistance.^7^ Therefore, novel antimicrobial treatment approaches for pathogenic multidrug-resistant bacteria are of vital global importance.^10^

The use of phages for the treatment of bacterial infections has a long history and may be an important tool in bacterial treatments in the future. First identified by Felix d’Herelle in 1917, they were used to treat dysentery among other bacterial infections with some reported success.^11,12^ However, research and use of bacteriophage therapies declined during World War II, largely driven by the discovery of penicillin.^13^ Recent developments have brought phage therapy back to the fore, such as a three-phage cocktail being used to treat disseminated *Mycobacterium abscessus* in a 15 year old patient with cystic fibrosis.^14^

For a phage to be efficacious in treating bacterial infections, certain characteristics are required including the ease of isolation and propagation, useful host range, and the absence of genes expressing proteins toxic to the patient.^15^ Phages often show an extremely narrow host range, thus requiring the development of phage cocktails to effectively treat bacterial infections. The host range of phages isolated from nature can differ depending on the assay used.^16,17^ It is often therefore problematic to balance the ability of the phage cocktail to treat an infection against the regulatory approval required for such mixtures.^15^

These issues were the motivation for engineering well-known and characterized phages to target alternative host bacteria rather than the traditional natural isolation approach. Characterized phages mean their host ranges are better defined and they are less likely to lyse nontarget bacterial strains, thus improving the likelihood of regulatory approval. A limitation of this area of research is that it has often been restricted to lytic phages and the hosts in which they propagate. This involves infecting a host with multiple phages of interest and selecting mutants able to propagate in the host, which can be laborious.^18,19^

Synthetic biological approaches have added an additional impact to the development of phage-based therapies,^20^ allowing for the modification of phage specificity through the expression of alternative tail fibers.^21,22^ Improvements were made by Ando et al., who swapped fragments of tail genes between phage relatives to extend the host range of the engineered phage. Their approach requires reconstruction of the phage genome in a yeast artificial chromosome (YAC) in which mutations are incorporated through a mechanism much like Gibson assembly^23^ and then reactivating the phage life cycle through transformation into bacteria. They identified that gene 17 (the tail fiber gene) was the primary host determinant and was thus able to extend T7 tropism to several non-*Escherichia coli* bacterial strains.^24^ However, this approach remained dependent on the propagation of phage and the picking of resultant plaques from mutants.

Propagation in a new host is a complex task and is dependent on multiple steps. These steps include adsorption, injection of DNA, replication, and cell lysis in addition to other host-dependent factors such as enzyme requirements. Yosef et al.^25^ suggested that to vastly extend the host range of a phage with speed and ease, one should focus on transduction. Transduction requires many fewer steps, thus simplifying the complex relationship between the host and phage. This simplification extends the host range of the phage due to the reduced number of limiting factors. The ability of phage therapeutics to self-replicate within a host cell population is often described as an advantage over traditional antibiotic treatments. However, concern over this has been raised, as this replication may cause side effects like tumor lysis syndrome due to the release of endotoxins.^26−28^ Therefore, a system that allows for the transduction but not the replication of the wild-type phage is a promising way forward. Using this principle to develop phage particles with hybrid tail fiber and spike genes, the host tropism could be increased to include hosts regardless of the phages’ ability to propagate within them. Fifteen hybrid T7 particles were thus produced through the expression of different tail genes *in trans* and were tested in 12 different hosts. The results were stark, as transduction was shown in all bacterial strains tested, compared to wild-type T7 only transducing five strains. Compared to the work by Ando et al. described above, this study extended the host range to include bacterial strains, such as *Klebsiella* and *Salmonella*, that would not have been possible in the previous approach as these strains do not support T7 propagation.^25^ Of note, however, this study required the deletion of all tail genes in the donor phage and so genes 11, 12, and 17 had to be supplied *in trans* for a complete phage particle to be produced.

Other approaches for extending the host range of phage have also been investigated, such as using random sequences in receptor binding proteins,^29^ BRED, a highly efficient method for recombineering through directed mutagenesis of phage genomes,^30^ and CRISPR-Cas9 systems could be utilized.^31,32^

**Figure 1:**
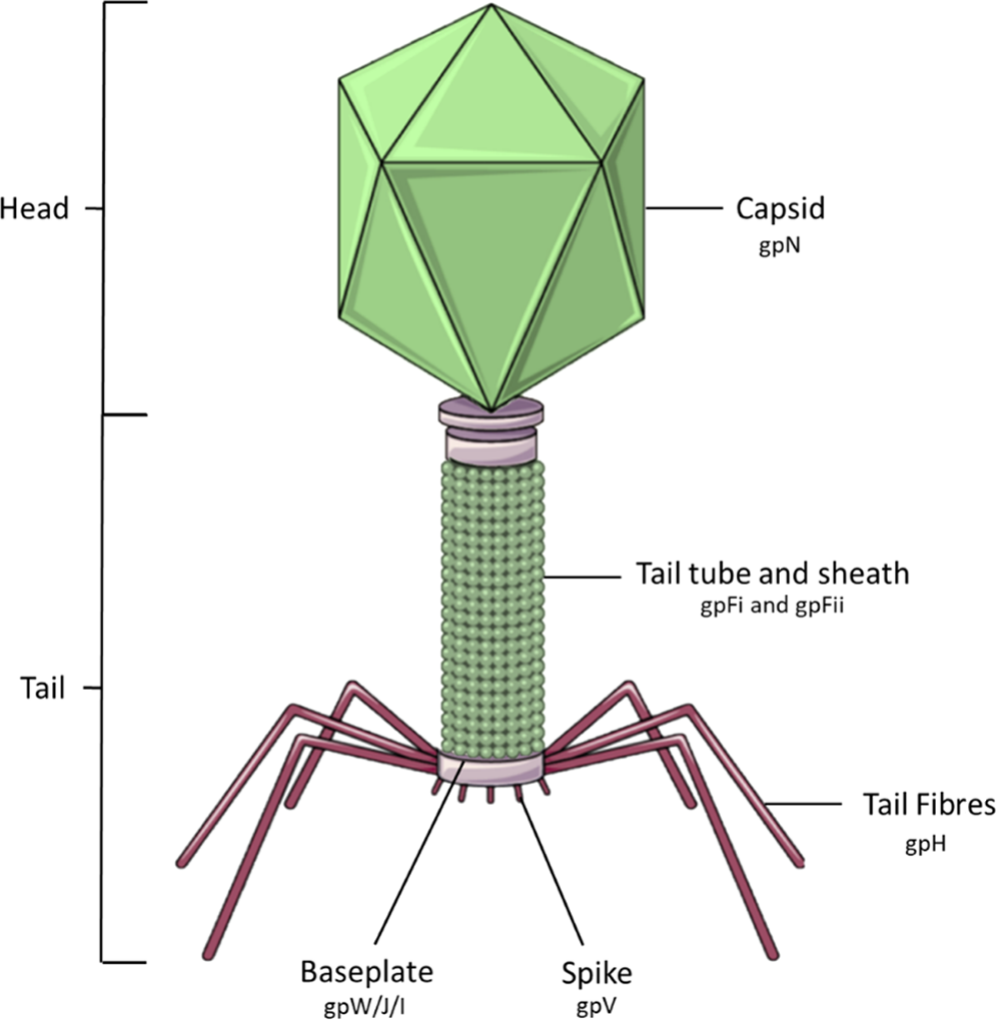
Structure of P2 bacteriophage labeled with the main structural genes.

Here, we develop an approach using phage P2 (Figure 1) for manipulation and the incorporation of chimeric tail fibers. P2 is a temperate phage first isolated in 1951 by Bertani.^33^ P2 binds and lyses *E. coli* via tail fiber attachment to lipopolysaccharides (LPS) on the bacterial membrane. The double-stranded DNA genome, of around 33 kb, is packaged into the icosahedral capsid. The capsid is mostly made up of a major capsid precursor, gpN, with accessory proteins gpO and gpL acting as scaffold and completion proteins, respectively. The tail fibers are made from gpH, with gpG required for assembly. The tail fibers (gpH) and the spike (gpV) have been shown to mediate the adsorption and infection of P2 into a host,^34^ although the tail fibers alone are used to bind to the target first, while the spike subsequently binds to other receptors in the bacterial membrane to strengthen the binding.^35^

P2 phage is highly permissive to genetic manipulation and has naturally formed fusions through a horizontal transfer with tail fibers from other bacteriophages to extend its host range in the past.^35^ Additionally, previous work in this area using T7 has required the modification of three tail genes.^25^ We sought to evaluate whether the editing of a single gene, the tail fiber gpH, would be sufficient for retargeting P2, thus simplifying the procedure for extending the host range.

We show that genetic modification of the P2 tail fiber genes can produce phage particles exhibiting significant adsorption and transduction to *Salmonella* bacterial cells without the need to culture pathogenic strains. Our study provides the first evidence that engineering the P2 tail fiber can extend the host range toward novel proteinaceous receptors, thus opening new potential applications for “designer bacteriophage” based on phage P2 in biotechnology fields. This work could also add to the growing use of designer phage to transduce bacterial strains with a synthetic DNA “pay-load” to kill and lyse the cells.

## 2 Materials and Methods

### 2.1 Strains and Plasmids

The strains and plasmids used in this study are listed in Table 1. All bacteria were grown in LB media at 37 °C at 200 rpm. Concentrations of antibiotics used are as follows: ampicillin (Amp) at 100 μg/mL, spectinomycin (Spec) at 50 μg/mL, kanamycin (Kan) at 50 μg/mL, and gentamicin (Gent) at 10 μg/mL.

**Table 1:** Bacteria, Phages, and Plasmids Used in This Study

bacterial strains	features	source
*E. coli* BW25115		Keio Collection Parental Strain, CGSC^36^
*E. coli* C1a	propagation strain for P2	(37)
*E. coli* Δ*rep*	deficient in ATP-dependent DNA helicase Rep, P2-immune	Keio Collection, CGSC; strain JW5604-1^36^
*E. coli* BW25115 ΔwaaC	KanR	Keio Collection, CGSC; strain JW3596-1^36^
*E. coli* subcloning efficiency DHα	chemically competent; SpecR	Invitrogen (ThermoFisher no. 18265017)
*Salmonella typhimurium* SL3261	KanR; attenuated strain	(33)

phage	features	source
P2_vir1_	lytic-only variant of P2 bacteriophage	(38)
S16	*Salmonella-*infecting lytic phage	(39)

cosmids	features	source
PKGB4 (PAJ693)	KanR	(38)
gpH-opt (PAJ694)	expresses P2-gpH and gpG codon optimized; GentR	designed by authors and synthesized by Genewiz
pUC-IDT-PK2 (PAJ695)	cosmid with longer cos region; AmpR	designed by authors and synthesized by Genewiz
pOmpC (PAJ659)	expresses *Salmonella* variant of OmpC gene; AmpR	designed by authors and synthesized by Genewiz
Lit2C (PAJ696)	first iteration of design based on previously described gene truncation points^39,40^ of P2-S16 tail fiber chimera; GentR	this study
HomA (PAJ697)	first iteration of the homology-based design of P2-S16 tail fiber chimera; GentR	this study
Lit2C-PK2 (PAJ698)	second iteration of the design based on previously described gene truncation points^39,40^ of P2-S16 tail fiber chimera with extended cos region; GentR	this study
HomA-PK2 (PAJ699)	second iteration with extended cos region; GentR	this study

### 2.2 Bacteriophage Preparation

The P2_vir1_ phage stock was produced from adapted previously published protocols.^41,42^ The full protocol is available in the Supporting Information. The production strain was *E. coli* C1a. Phage S16 was produced as per previously published protocols.^43^

### 2.3 Plaque Assay with P2_vir1_ Lysates

A bacterial culture grown overnight was refreshed, and growth was continued to OD_600_ = 0.2–0.3. Serial dilutions of the lysate were prepared. The culture (0.3 mL) and diluted lysate (0.1 mL) were combined with CaCl_2_ to a final concentration of 5 mM and incubated at 37 °C for 10 min. Top agar prewarmed to 42 °C was added (3 mL), mixed by inversion, and poured into LB agar plates supplemented with 5 mM CaCl_2_, swirling by hand to ensure even coverage. Plates were incubated at 37 °C overnight
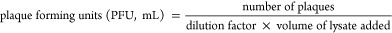
1

### 2.4 Transduction Assay with P2_vir1_ Lysates

A bacterial culture was grown overnight, supplemented with CaCl_2_ to a final concentration of 5 mM and l-arabinose to 0.1% (if inducing chimeric tail fiber expression) and shaken for an additional 15–30 min at 37 °C. The culture (0.2 mL) was combined with a lysate (0.2 mL) at an MOI of 0.1 and incubated for 20 min at 37 °C. The CaCl_2_ was chelated by adding a 1:1 volume of 1 M sodium citrate. Top agar prewarmed to 42 °C was added (3 mL), mixed by inversion, and poured into LB agar plates supplemented with appropriate antibiotics. Plates were incubated overnight at 37 °C
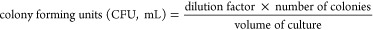
2

Transduction efficiencies were calculated using eq 3, using data from both plaque assay and transduction assays (eqs 1 and 2)
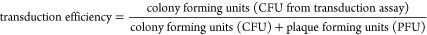
3

### 2.5 Design, Creation, and Cloning of P2-gpH/S16-gp37 Chimeras

Two chimeric tail fiber designs were created using different fusion points between the tail fiber genes of the two phages, P2 and S16. First, a design based on previously described gene truncation points for the P2-gpH and the S16-gp37 genes was used.^39,40^ The second approach used a homology region identified by an alignment between the amino acid sequences of the tail fiber genes at 564–599 of gp37 to amino acids 481–516 of gpH. The homology covered an area of 17 amino acids, with a 76% identity. Regulatory elements including RBS sites, terminators, and promotors were gathered from Scholl and Williams,^40^ or the iGEM registry of standard biological parts.^44^

Designs were synthesized as dsDNA by Genewiz, which were amplified with Q5 high-fidelity DNA polymerase as per the manufacturers’ procedures. PCR products were treated with *Dpn*I (R01765, NEB) and purified using a Qiagen QIAquick PCR purification kit. Cloning was completed using the Gibson assembly method, as described in Gibson et al.,^23^ and the resultant plasmids were transformed into subcloning efficiency DH5α competent cells (Invitrogen). Sanger sequencing confirmed plasmid sequences.

### 2.6 Phage Pulldown

Overnight cultures of *S. typhimurium* SL3261 to be tested were adjusted to an OD_600_ = 1.0, equaling ∼10^9^ colony-forming units/mL using LB containing 0.02% Tween-20. Phage lysates were added to an MOI of 0.01 and mixed at room temperature for 10 min. After centrifugation at 13 000*g* for 2 min, the supernatant containing the unbound phage was collected and used to infect the relevant propagation strain for the phage using soft agar overlays. Equation 4 determined the adsorption ratio^39^
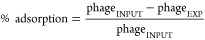
4phage_INPUT_ = phage from the control reaction, phage_EXP_ = PFU from test reaction.

### 2.7 Statistical Analyses

Data values from several repeats were averaged, and standard deviations were calculated. One-sided *T*-tests with a confidence level of 95% were performed.

## 3 Results

### 3.1 Evaluating Transduction Efficiency of Cosmids

**Figure 2:**
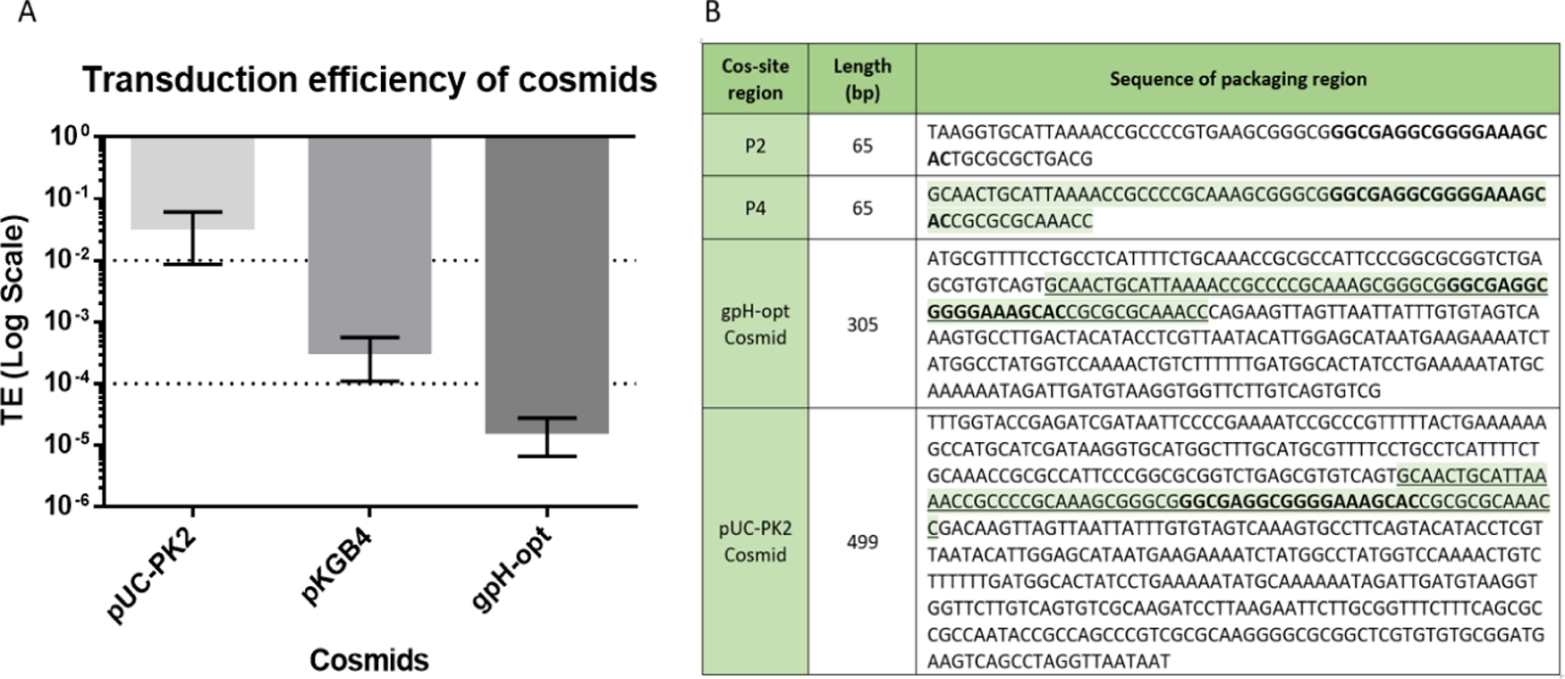
Differences in transduction and sequence of cosmids. (A) Transduction efficiencies of cosmids (CFU/mL performed on Δrep bacterial strain); an additional “no plasmid” control was carried out, which showed a transduction efficiency of 0 and is not graphed here. Transduction efficiency was calculated through eq 3 after plaque assays and transduction assays (using eqs 1 and 2). (B) Sequencing of packaging signals. The cos site is in bold, and the P4 packaging region is highlighted in pale green and is found in both cosmids, instead of the P2 cos-site region.

The transduction efficiency of P2-cosmids available was evaluated to generate standardized baseline levels for P2_vir1_ transduction in *E. coli*. This was for the comparison and identification of the cosmid with the highest transduction efficiency. The transduction efficiency of three P2-cosmids was tested (Figure 2A). P2-cosmid PAJ695 demonstrated the highest transduction efficiency of 0.035, compared to the gpH-opt plasmid, which demonstrated the lowest efficiency of 1.7 × 10^–5^.

Due to the vast differences in the transduction efficiencies, the packaging signal regions of these cosmids were investigated further. The packaging signal regions were found to vary in length, which may contribute to their different transduction abilities (Figure 2B).^41^ The packaging signal from PKGB4 is composed of a 1 kb fragment of the P4 phage genome including the cos site.^41,45^ P2 and its satellite phage P4 do not generally share homology, except for the highly conserved 55 bp long cos site.^46,47^ The pUC-IDT-PK2 cosmid has longer regions flanking the cos site, which might contribute to its improved transduction efficiency; thus these flanking regions to the cos site are also important in designing cosmids for high transduction efficiency.

### 3.2 Chimeric Tail Fiber Designs

**Figure 3:**
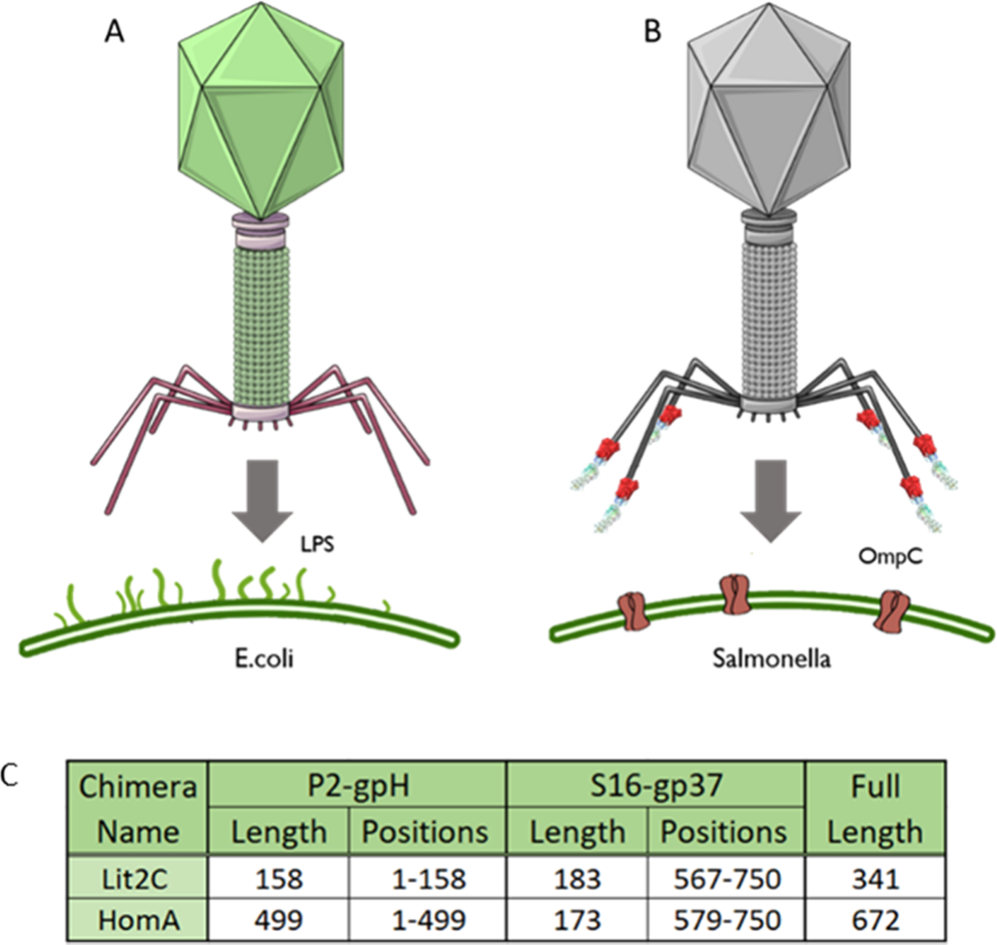
Native binding of the parental phage used in this study and the design of chimeric tail fibers. (A, B) Native binding capabilities of the phage used in this study. (C) A table showing the relative length of each protein sequence incorporated into both designs and the positions of these amino acids in the full protein sequence (i.e., N-terminal section from gpH and a C-terminal section from gp37). Additionally, the full length of amino acids of each chimera is shown.

Two chimeric tail fiber designs were considered, using the tail fiber genes from P2 and a secondary phage, S16, which infects and lyses *Salmonella* bacteria (Figure 3A). The aim of this was to retarget P2 toward the protein receptor, OmpC, found in *Salmonella*. This target was chosen as a fully functional truncated S16 tail fiber had been previously described by Marti et al.,^39^ allowing for a shorter chimera, in addition to growing concerns about antibiotic-resistant *Salmonella* species.^48^ The relative lengths of the genes from both phages in these designs are described in Figure 3C.

### 3.3 Evaluation of ΔwaaC-pOmpC Cell Line for P2_vir1_ Resistance

**Figure 4:**
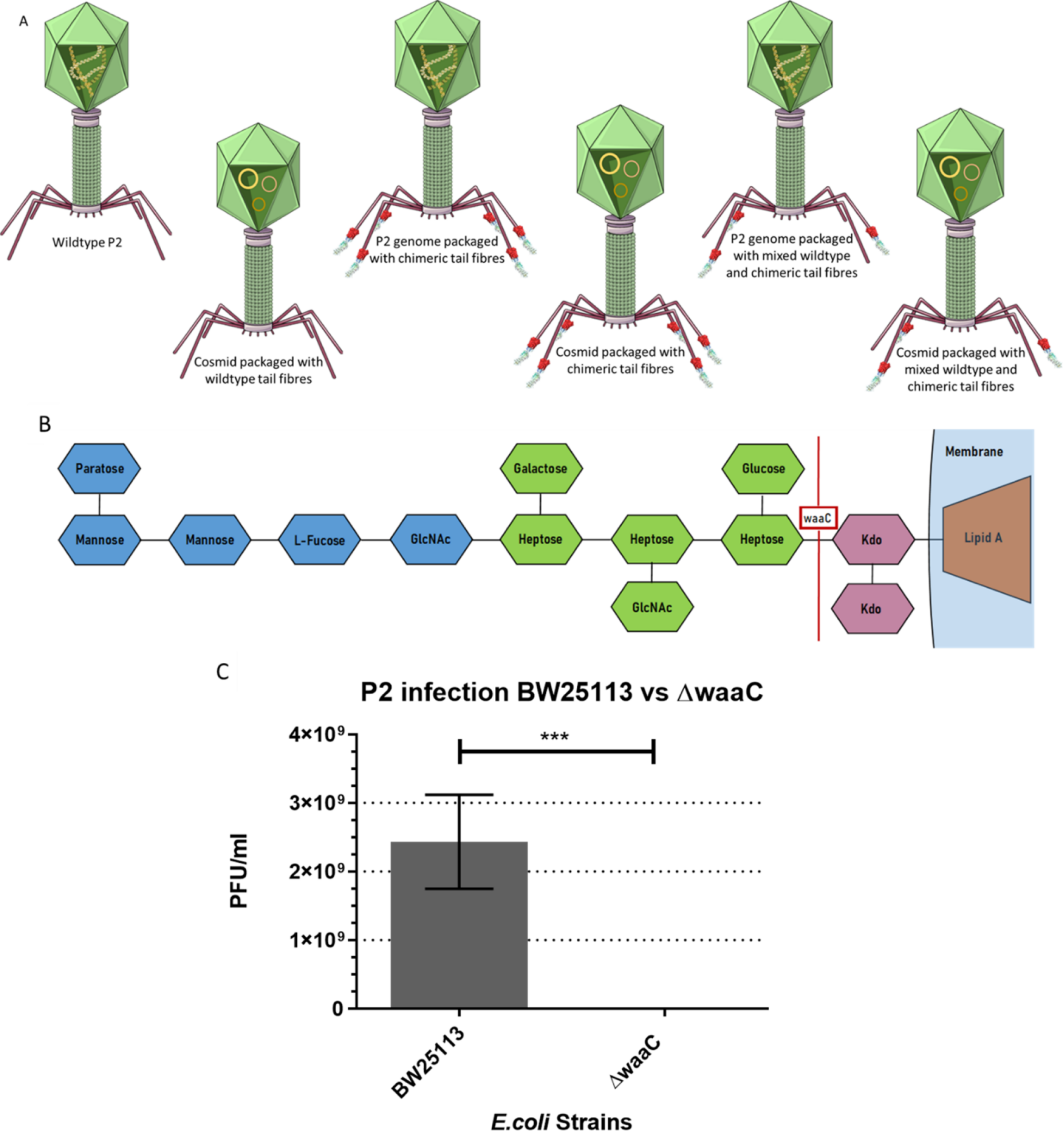
Validation of ΔwaaC *E. coli* cells being resistant to P2_vir1_ infection. (A) Diagrams showing the progeny produced from P2 infection in bacterial cells harbouring a chimeric tail fiber cosmid, a wild-type P2_vir1_ phage, virions with the chimeric tail fiber cosmid packaged with wild-type tail fibers, virions with wild-type genome and chimeric tail fibers, virions with cosmid packaged and chimeric tail fibers, and virions with either the genome or cosmid packaged that are expressing a mixture of tail fibers. (B) Structure of LPS on bacterial cells showing that the removal of the waaC gene would cause an extremely truncated LPS. (C) A bar graph showing a significant reduction in PFU/mL in ΔwaaC cells compared to BW251113. ***P ≤ 0.001 (using eq 1).

We sought to evaluate the transduction efficiency of phage binding through the alternatively targeted receptor, OmpC, and not binding the original receptor, LPS. In the production of a lysate for testing, up to six types of progeny phages could be produced. One strategy to differentiate between binding capabilities of the phage lysate is antibiotic selection via the antibiotic resistance gene expressed on the chimeric tail fiber cosmid. This limits the possible positives in a transduction assay to only phage particles, with the cosmid packaged in the capsid (Figure 4A). It is also important to note that due to the method of lysate production, some phage progeny may be present with a mixture of tail fibers. As a bacterial strain containing the cosmid of interest infected with the P2_vir1_ donor phage, both types of tail fiber genes are available in the host. This means that the phage could be produced with different numbers of wild-type and chimeric tail fibers expressed on one phage particle. The likelihood of these types of progenies being produced is difficult to estimate. However, due to the protocol we have implemented, progeny with tail fiber mixtures and wild-type genome packaged in the capsid will not confer the required antibiotic resistance to rescue bacterial cells in transduction assays. Those progeny with tail fiber mixtures and the cosmid packaged will, however, confer resistance and so these will be counted in any transduction efficiency result.

As has been proposed with phage P22, three trimeric tail fibers could be sufficient to ensure committed adsorption^49^ and so perhaps a virion with at least three homo-trimeric tail fibers could be enough to guarantee committed adsorption here. If all rearrangements are assumed to be equally probable, then half of all progenies would be able to develop committed adsorption on all of the homo-trimeric rearrangements.

Since LPS is present in most bacterial cells, we wanted to evaluate bacterial strains genetically deficient in LPS, which ought to be resistant to P2_vir1_ infection. There are many types of LPS mutants available; however, ΔwaaC mutants provide the most severe truncation of the LPS molecule^50^ (Figure 4B) and thus potentially the most decreased P2_vir1_ absorption. ΔwaaC cells were evaluated for their resistance to P2_vir1_ infection and compared to BW25113, which expresses full-length LPS using plaque assays.

Plaque assays demonstrated that ΔwaaC is indeed resistant to P2_vir1_ infection, with no plaques detected at a range of lysate concentrations from neat to 10^–8^ dilution (Figure 4C), which supports previously published findings.^51^ These ΔwaaC cells were then transformed with the OmpC plasmid, creating the ΔwaaC-pOmpC strain, which is resistant to P2_vir1_, but may be sensitive to infection by progeny expressing chimeric tail fibers. Therefore, the ΔwaaC-pOmpC strain, in conjunction with antibiotic selection, will select for progeny displaying chimeric tail fibers and cosmid DNA packaged in the capsid.

### 3.4 Testing the Packaging Efficiency of the Chimeric Tail Fiber Cosmids and Improvement

**Figure 5:**
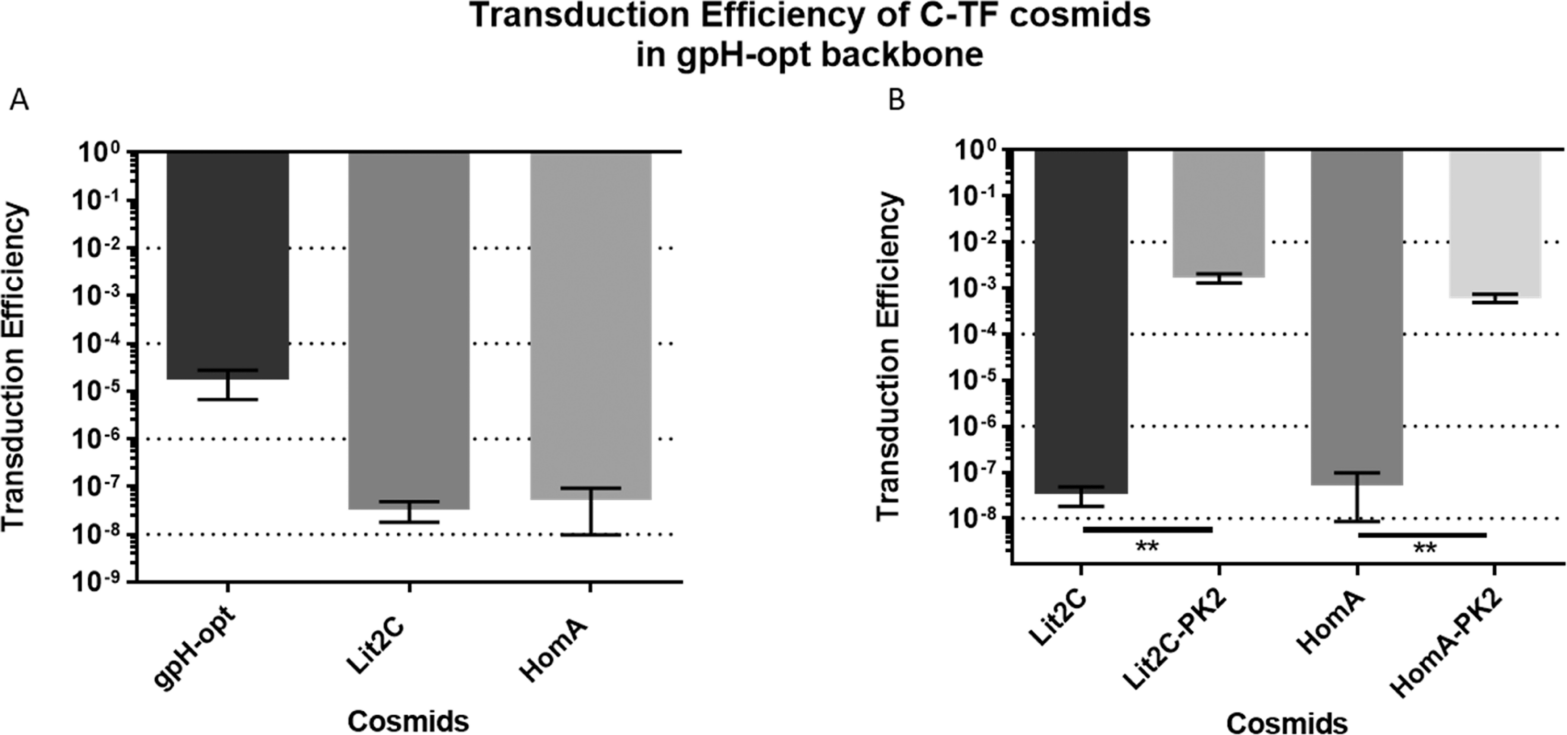
Transduction efficiency of chimeric tail fiber designs. “No plasmid” control was carried out, which showed a transduction efficiency of zero and it is not shown. (A) Showing the parental cosmid and first round of designs. The transduction efficiencies were calculated using eq 3 from PFU assays in strain C1a and CFU assays using strains Δrep. (B) Showing the designs updated with the pUC-IDT-PK2 packaging signal, which significantly improves transduction. Unpaired *T*-test, **P ≤ 0.01.

The chimeric tail fiber genes were first cloned into the gpH-opt cosmid, which showed low transduction efficiency previously (Figure 2A). However, the cloning reduced the transduction efficiency of gpH-opt cosmid further to 3.33 × 10^–8^ and 5.16 × 10^–8^ for Lit2C and HomA, respectively (Figure 5A). Efficiency was improved by replacing the packaging signal originally found in the gpH-opt plasmid, with the longer packaging signal found in pUC-IDT-PK2. This significantly improved transduction efficiencies of the chimeric tail fiber designs, ∼50 828× and 11 597× fold for the Lit2C and HomA design, respectively, and as such they were renamed Lit2C-PK2 and HomA-PK2 (Figure 5B).

### 3.5 Pseudotyped P2 Can Be Significantly Retargeted to *Salmonella* Via OmpC

**Figure 6:**
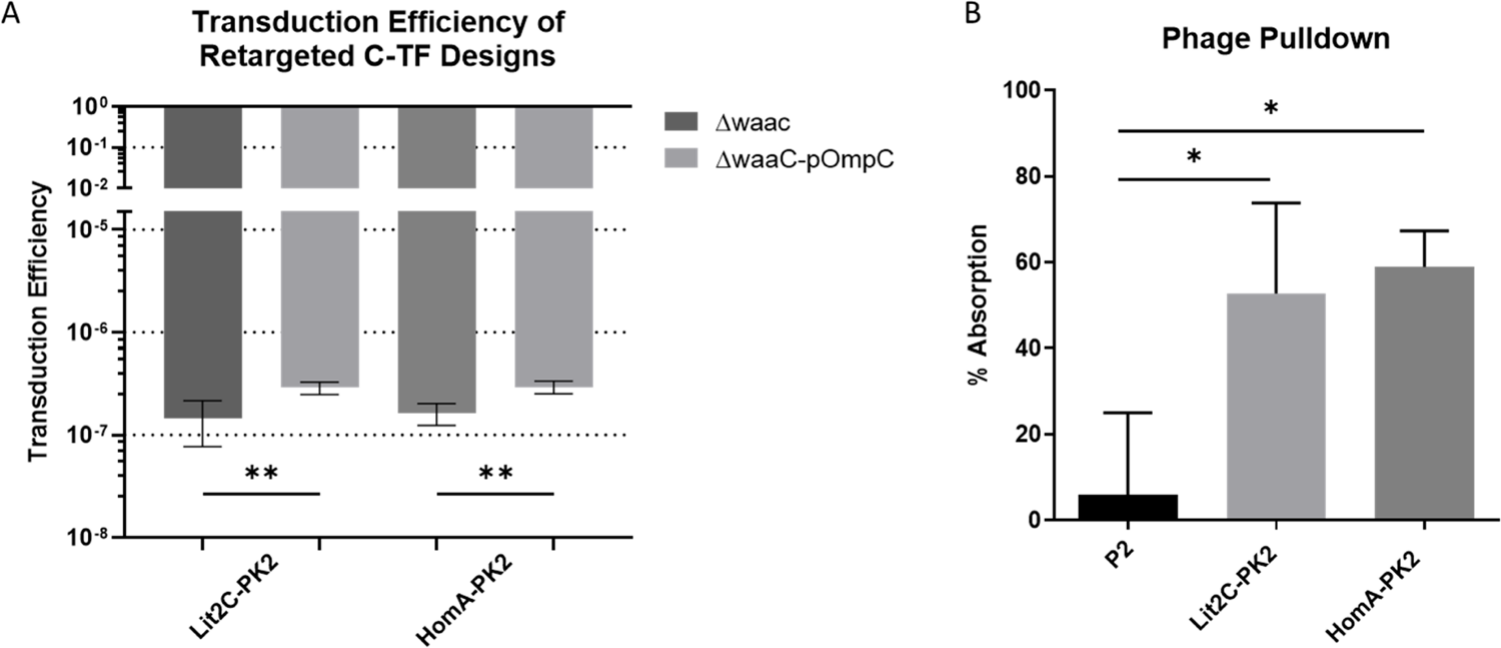
Chimeric tail fibers significantly retarget the P2_vir1_ phage toward protein receptor, OmpC. (A) Both Lit2C-PK2 and HomA-PK2 show successful and significant retargeting toward OmpC when infecting ΔwaaC-pOmpC compared to ΔwaaC. Parental gpH-opt cosmid was also tested in ΔWaac +/–pOmpC strains, with a transduction efficiency of 0, and hence is not graphed. (B) Phage adsorption to Salmonella strain, SL3261 (calculated using eq 4), significantly increased for the chimeric tail fiber (52.8% and 58.9%, respectively, for Lit2C-PK2 and HomA-PK2) designs compared to P2_vir1_ adsorption (5.9%). Unpaired *T*-test, *P ≤ 0.05, **P ≤ 0.01.

The phage progeny produced in this system with the chimeric tail fibers was tested for retargeting ability in the ΔwaaC +/–pOmpC strains. Transduction efficiency was significantly increased for both chimeric phage particles between the strains, ∼2× fold and 1.8× fold for Lit2C-PK2 and HomA-PK2, respectively (Figure 6A).

Both chimeric phage particle lysates were tested in *S. typhimurium*, SL3261, which expresses the OmpC receptor.^52−54^ Using phage pulldown assays, we showed that both these phage lysates bound significantly to SL3261 compared to P2_vir1_ (Figure 6B).

## 4 Discussion

Synthetic bacteriophages show promise as targeted therapeutics. Specifically, a designer phage that is targeted toward a protein receptor of interest and that allows for the transduction of foreign DNA into bacterial cells would be a useful addition to the antimicrobial toolbox.

Here, we develop one such method based on the P2 phage. We designed two chimeric tail fiber designs, which, when supplied *in trans*, show evidence of retargeting away from P2s natural LPS-mediated means of cellular entry and toward a novel protein receptor. These phage progeny particles show significant absorption to both *Salmonella* and *E. coli* expressing the *Salmonella* version of OmpC. Importantly, with the tools we had available, we were unable to examine whether these chimeric phage particles were able to transduce Salmonella as well as the *E. coli* strain expressing OmpC. This is due to the mixture of phage progeny produced in the lysate (as shown in Figure 4A). The *Salmonella* strain SL3261 expresses both LPS (the wild-type P2 receptor) and OmpC (the target); therefore, any phage progeny with the cosmid packaging in the capsid would be able to potentially transduce the bacteria in any transduction assays. Two methods could be employed to overcome this issue in further work. First, a helper P2 phage with ablated tail fiber genes could be created^55^ to remove the contamination of wild-type tail fibers in the lysate. In this way, only phage progeny with cosmid packaged in the capsid and chimeric tail fibers expressed on the particle would be able to rescue the bacteria and form colonies in transduction assays. Second, we could create a “ΔwaaC” *Salmonella* strain with the purpose that those progenies with wild-type tail fibers would not infect as with the ΔwaaC *E. coli* strain, despite the contamination with the wild-type tail fiber genes in this system (Figure 4C). These methods would be able to further support the results presented in this paper and confirm if the results displayed here are truly due to chimeric retargeting of the P2 particle or a side effect of the P2 helper phage contamination in the lysates.

This study also highlights the importance of a longer packaging signal sequence for increased transduction efficiency (Figure 3). This demonstrates that both the fusion points between potential genes and the additional DNA flanking the packaging signal are critical, as these can enhance the transduction efficiency by over 1000-fold. The efficiency of packaging could be improved by using the re-engineered P4 phage.^55^

The use of two design approaches allowed for their comparison, which may prove useful in the design of future chimeric tail fibers. Both design approaches yielded significant results in the transduction efficiency of ΔwaaC-pOmpC and more importantly of *Salmonella* SL3261 in addition to phage pulldown studies (Figure 6). This information provides insights into the optimal design of chimeric tail fibers that a highly efficient packaging signal may be more important than fusion points between tail fiber genes of interest. We believe that the potential to use areas of homology between other phage tail fibers and gpH of P2_vir1_ would provide a relatively straightforward methodology to design chimeras without the painstaking work of interrogating the efficiency of binding at different fusion points.

Our work aims to further the use of P2 chimeric tail fibers in targeting and potentially treating pathogenic bacterial strains. In further work, it would be interesting to use the additional capacity of the P2_vir1_ capsid to encode therapeutic payloads. The capsid has a capacity of 33 kb, which based on these chimera designs would allow for the inclusion of ∼26–27.5 kb of additional DNA. The Lit2C-PK2 design would allow for an additional 1.5 kb of cargo compared to the HomA-PK2. The ability to retarget a phage quickly and effectively toward a new bacterial strain and efficiently transduce therapeutic payloads would be of great importance in the fight against antibiotic-resistant bacteria.

Previous work in this area utilized a captured phage genome in a yeast artificial chromosome for easy introduction of mutants; however, this method requires the propagation of the resulting phage particles and so is limited to hosts that support that.^24^ Yosef et al.^25^ were the first to describe focusing on the transduction of bacterial strains instead of propagation, and thus extending to many more hosts than previously would have been possible. Their approach also supplemented chimeric tail fibers *in trans* for T7 and yielded a significant host range extension. However, as they used T7 phage, three tail genes (gene 11, 12, and 17) were required to be ablated from the donor phage and then supplemented in their mutant forms *in trans*.^25^

Here, we were able to show promising data on retargeting P2 through the engineering of a single gene, the tail fiber gene of P2, gpH. This greatly improves the speed of the chimeric tail fiber design as it reduces the number of genes for manipulation to a single gene. We also focused on the transduction of phage particles, allowing for the selection of particles expressing the chimeric tail fibers with the cosmid packaging in the capsid in one transduction assay step. The P2 donor phage used still retains a fully intact genome, meaning that the step of engineering the phage genome itself is not required. Engineering phage genomes can be challenging due to the transience of phage DNA. Homologous recombination is commonly used; however, this is inefficient since lytic phages often degrade resident DNA upon cell entry, coupled with the lack of selectable marker, so not requiring this step is therefore a benefit,^56,57^ although new techniques such as BRED,^30^ MAGE,^58^ and CRISPR-Cas9 systems could be utilized to overcome this.^31,32,59^ Further work into the ablation of the tail fiber gene in the donor phage will be important for future applications,^55^ particularly when aiming for regulatory approval for use as a therapeutic.

In conclusion, the present study develops P2 as a useful phage in the arena of phage therapeutics. We generated chimeric tail fibers presented on phage P2, which show promising data on retargeting the phage toward a protein receptor that is expressed in *Salmonella*. This gives the proof of principle that this approach with P2 could be used to effectively retarget phage particles toward pathogenic bacterial strains without the need to culture them. The engineering of phage able to adsorb through proteinaceous receptors allows using alternative hosts to optimize them, opening the way toward the engineering of phage against bacteria from their genomic knowledge of their surface proteins.

## Supporting Information Available

Plasmid maps and full sequences of Lit2C-PK2 and HomA-PK2; and plate images of plaque assay comparing P2 infection in ΔwaaC and BW25113 bacterial strains (−3 to −8) (PDF)
