# Supplementary material for: Pseudotyping Bacteriophage P2 Tail Fibers to Extend the Host Range for Biomedical Applications
Source: ACS Synth Biol. 2022 Sep 9;11(10):3207–15. doi: 10.1021/acssynbio.1c00629 (PMC9594776; doi:10.1021/acssynbio.1c00629)
Supplement: Supplementary file 1 — sb1c00629_si_001.pdf [file sb1c00629_si_001.pdf]

Supplemental information

Supplemental Methods

Protocol 1: Preparation of P2 Bacteriophage Lysates

Materials required:

- LB
- LBA (LB Agar)
- 1M Calcium Chloride ($CaCl_2$)
- P2 Bacteriophage
- 0.1M Magnesium Sulphate ($MgSO_4$) Chilled
- 1M Magnesium Chloride ($MgCl_2$)
- Glucose (40% solution)
- EDTA (Ethylenediaminetetraacetic Acid)
- Chloroform
- 1M Sodium Citrate

1. Create an Over Night Culture (ONC) of bacterial cells of interest at 37°C
2. Refresh the ONC into 10ml fresh LB supplemented with 2mM $CaCl_2$
3. Grow at 37°C and 220 rpm until it reaches an OD_{600} = 0.2-0.3
4. Add an appropriate volume of P2vir1 to satisfy an MOI of 0.1
5. Let it stand for 10min at 37°C
6. Centrifuge at >3000G for 10mins and discards the supernatant
7. Wash in 10ml of chilled 0.1M $MgSO_4$
8. Repeat steps 6-7 two or three times depending on the application and then centrifuge at >3000G for 10mins
9. Discard supernatant and refill with 10ml LB
10. Add $CaCl_2$ to create a final concentration of 5mM, $MgCl_2$ to a concentration of 16 mM and an appropriate volume of Glucose to make the solution 1% Glucose
11. Shake at 180-200rpm at 37°C for 1hr and then add EDTA to a final concentration of 10mM
12. Continue to shake at 250rpm at 37°C until lysis is obvious (appearance of filaments or solid particles)
13. Add 1ml chloroform and incubate at room temperature for 30mins, slowly shaking
14. Centrifuge at >3000G at room temperature for 10mins
15. Collect the supernatant and filter through a 0.2μM membrane
16. Store at 4°C

The titre is normally checked on a appropriate bacterial strain and in the region of 10^8 . This protocol can be used to make complete or transducing phage particles.

Supplemental Figures and Tables

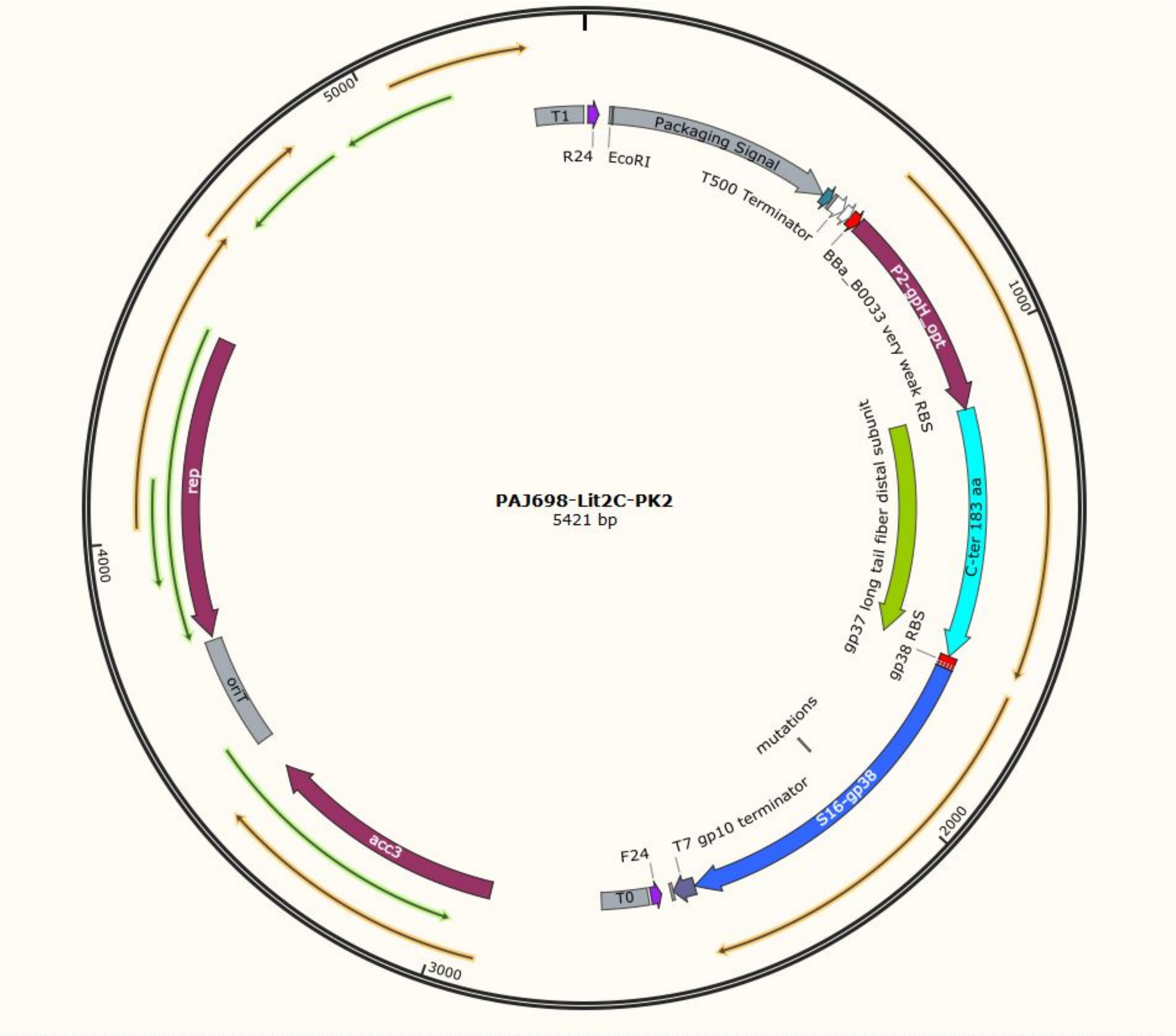

Supplemental Figure 1 – Plasmid Map of Lit2C-PK2. Full DNA sequence provided as PAJ698-Lit2C-PK2.dna

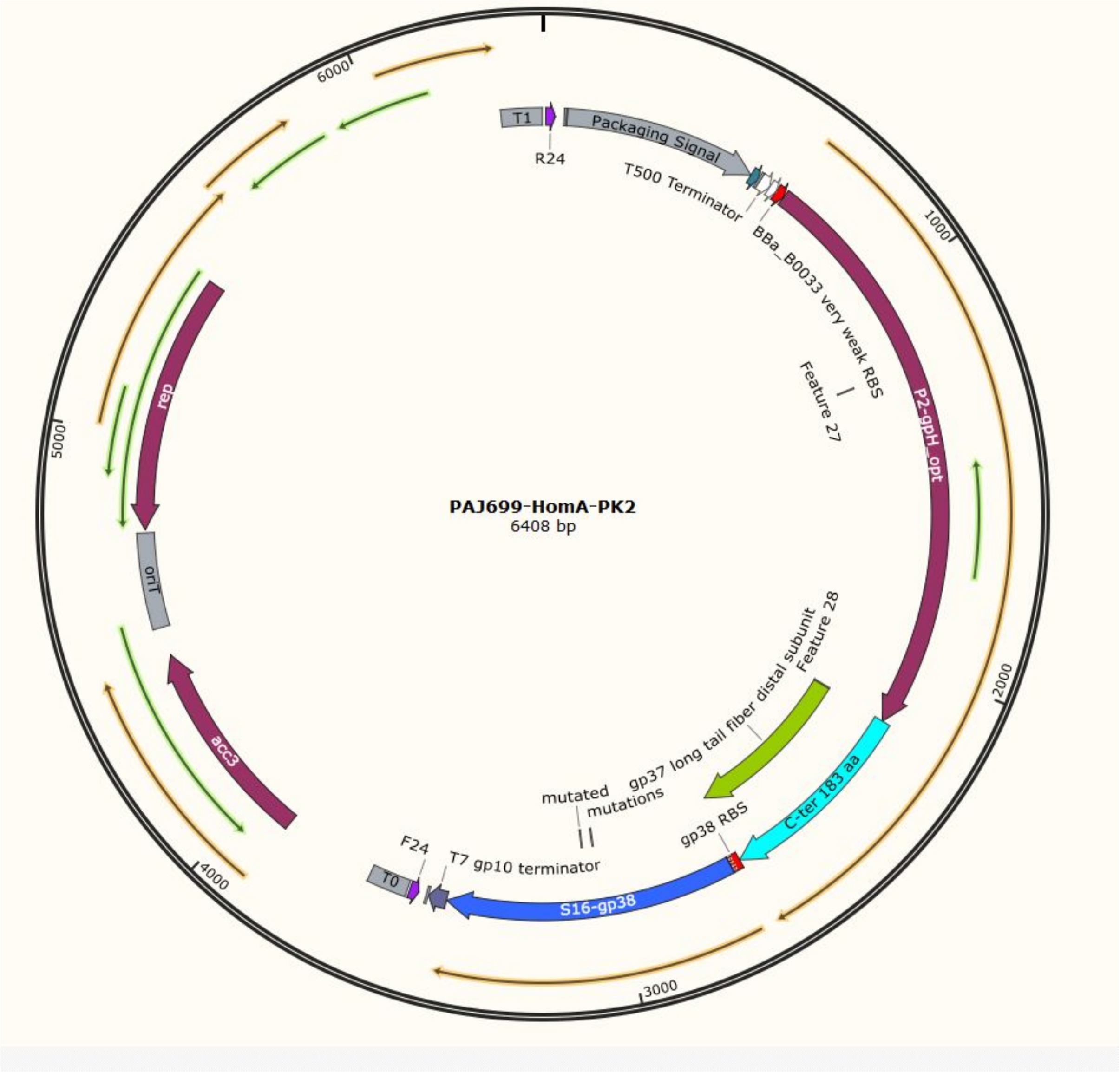

Supplemental Figure 2 – Plasmid Map of HomA-PK2. Full DNA sequence provided as PAJ699-HomA-PK2.dna

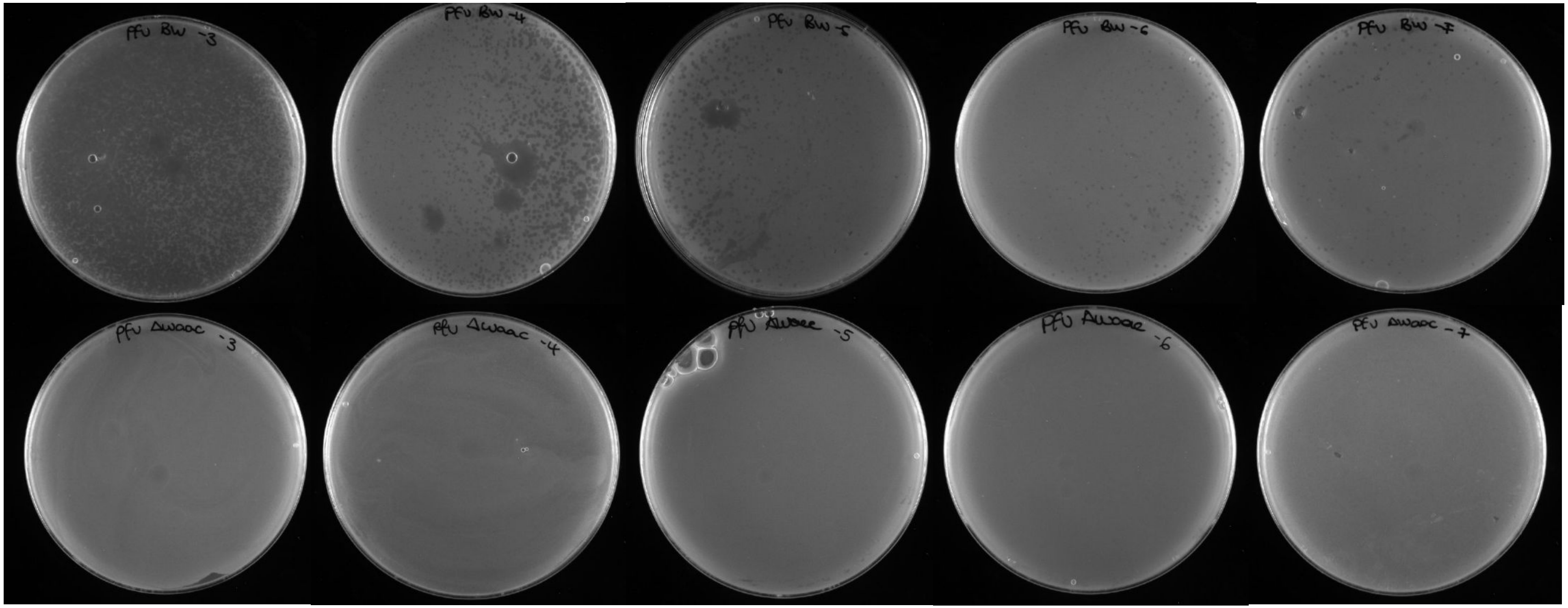

Supplemental Figure 3 – Plates showing plaque assay in BW25113 and delta-waac bacterial strains (top and bottom rows respectively). BW25113 (top) are susceptible to P2vir1 infection, whereas delta-waaC (bottom) are not susceptible as no plaques are formed. Dilutions show from 10^{-3} -10^{-8} (left – right).

Supplemental Table 1 - Raw data of Figure 6A - Transduction Efficiency in ΔwaaC +/- pOmpC of chimeric tail fibres lysates

Lit2C-PK2 (ΔwaaC)	Lit2C-PK2 (ΔwaaC-pOmpC)	HomA-PK2 (ΔwaaC)	HomA-PK2 (ΔwaaC-pOmpC)
2.10E-07	3.02E-07	1.89E-07	3.10E-07
2.18E-07	3.20E-07	1.30E-07	3.15E-07
2.00E-07	3.06E-07	1.15E-07	3.25E-07
7.50E-08	2.10E-07	1.40E-07	3.15E-07
8.00E-08	2.90E-07	2.06E-07	2.15E-07
9.50E-08	2.95E-07	1.98E-07	2.80E-07

Supplemental Table 2 - Raw Data of Figure 6B - Phage adsorption to Salmonella of chimeric tail fibre lysates

P2	Lit2C-PK2	HomA-PK2
25	50	66.66667
5.882353	33.33333	50
-13.2075	75	60
